# Benefits of the Cybathlon 2020 experience for a prosthetic hand user: a case study on the Hannes system

**DOI:** 10.1186/s12984-022-01046-y

**Published:** 2022-07-04

**Authors:** Giulia Caserta, Nicolò Boccardo, Marco Freddolini, Giacinto Barresi, Andrea Marinelli, Michele Canepa, Samuel Stedman, Lorenzo Lombardi, Matteo Laffranchi, Emanuele Gruppioni, Lorenzo De Michieli

**Affiliations:** 1 Rehab Technologies, Istituto Italiano di Tecnologia, Via Morego, 30, 16163 Genova, Italy; 2 Department of Informatics, Bioengineering, Robotics and Systems Engineering, University of Genova, Viale Causa, 13, 16145 Genova, Italy; 3 Centro Protesi INAIL, Istituto Nazionale Per L’Assicurazione Contro Gli Infortuni Sul Lavoro, 14, Via Rabuina, 40054 Bologna, Italy

**Keywords:** Myoelectric prosthesis, Upper limb prosthetics, Cybathlon, Transradial amputees, Functionality, Embodiment, User experience, Case report

## Abstract

**Background:**

Cybathlon championship aims at promoting the development of prosthetic and assistive devices capable to meet users’ needs. This paper describes and analyses possible exploitation outcomes of our team’s (REHAB TECH) experience into the Powered Arm Prosthesis Race of the Cybathlon 2020 Global Edition, with the novel prosthetic system Hannes. In detail, we present our analysis on a concurrent evaluation conducted to verify if the Cybathlon training and competition positively influenced pilot’s performance and human-technology integration with Hannes, with respect to a non-runner Hannes user.

**Methods:**

Two transradial amputees were recruited as pilots (Pilot 1 and Pilot 2) for the Cybathlon competition and were given the polyarticulated myoelectric prosthetic hand Hannes. Due to COVID-19 emergency, only Pilot 1 was trained for the race. However, both pilots kept Hannes for Home Use for seven weeks. Before this period, they both participated to the evaluation of functionality, embodiment, and user experience (UX) related to Hannes, which they repeated at the end of the Home Use and right after the competition. We analysed Pilot 1’s training and race outcomes, as well as changes in the concurrent evaluation, and compared these results with Pilot 2’s ones.

**Results:**

The Cybathlon training gradually improved Pilot 1’s performances, leading to the sixth place with a single error in task 5. In the parallel evaluation, both pilots had an overall improvement over time, whereas Pilot 2 experienced a deterioration of embodiment. In detail, Pilot 1, who followed the training and raced the Cybathlon, improved in greater way.

**Conclusion:**

Hannes demonstrated to be a valuable competitor and to perform grasps with human-like behaviors. The higher improvements of Pilot 1, who actively participated in the Cybathlon, in terms of functionality, embodiment and UX, may depend on his training and engagement in the effort of achieving a successful user-prosthesis interaction during the competition. Tasks based on Cybathlon’s ones could improve the training phase of a prosthetic user, stimulating dexterity, prosthetic integration, and user perception towards the prosthesis. Likewise, timed races or competitions could facilitate and accelerate the learning phase, improving the efficiency and efficacy of the process.

## Background

Losing a limb has devastating different consequences: the amputee is no longer able to perform his usual activities of daily living (ADLs), he is not completely autonomous and independent, and this results in a dramatic decrease of the quality of life [1]. The loss of an upper limb irreversibly alters the look and affective interactions of the amputee, causing severe repercussion such as social rejection, self-pity and low self-esteem. In addition, also the biomechanics of the body changes, trying to compensate the missing limb. This leads to the excessive use of the rest of the body or to incorrect body postures, which in turn produce extreme fatigue [2].

Typically, the main strategy for compensating a hand loss is using an artificial upper limb, a prosthesis. A prosthesis is, therefore, an assistive device which should become an essential element of the amputee’s daily life. Current prosthetic options range in both cosmetics and functionality, to satisfy a variety of user needs and lifestyles. The most advanced prostheses currently available are the so called “Myoelectric prostheses”. These devices use electromyographic (EMG) signals, generated from the contraction of the stump’s residual muscles, for the activation of the functional elements. However, even though they offer an increased functionality compared to the simpler options such as cosmetic or body-powered, there are still problems related to weight, cost, maintenance, reliability, and complexity of control [3].

Due to all these limitations, the device abandonment rate is still high, and a large part of the amputees’ population even prefers not to use any prosthesis at all during their daily life [4]. Decades of research and development (R&D) on bionic limbs suggest that the design of prosthetic hands requires accurate investigations about users’ needs, paying attention at improving user experience. Indeed, this may increase the technology acceptance of the prostheses. UX depends on many factors such as the control approach used to control the prosthesis [5], interactive training methods [6], embodiment stimulation [7], and biomimetic design strategies [8]. Furthermore, the development of a prosthesis capable to induce the embodiment and to be felt as a valid and natural substitution of the missing limb by the amputee, rather than a simple tool, is an important target as well.

However, the detachment between the communities of users, researchers, developers, and all stakeholders can become an obstacle for fertile improvements in prosthetic user experience. Thus, novel strategies for gathering these communities [9] around the same scope, possibly also promoting a proper representation of the user’s issues and needs, could be advantageous.

The Cybathlon competition for assistive technology have been conceived to reach such a result, reducing the gap discussed above [10]. This unique world championship proposes six different disciplines in which people with physical disabilities compete against each other with both commercial devices and research prototypes. Within this context, the Powered Arm Prosthesis Race is dedicated to people with transradial or more proximal arm amputation, or dysmelia, and equipped with arm prosthesis. Preparing for such a competition requires a careful attention to realistic tasks, increasing the ecological validity of the user performance assessed during the competition—potentially as in technology benchmarking [11]. On the other hand, training a prosthetic user for a Cybathlon challenge means for R&D teams the opportunity of exploit these tasks to explore novel solutions that will be extensively tested according to clinical protocols.

This paper presents the Cybathlon 2020 experience of our participating Pilot 1 with the novel prosthetic system Hannes. Furthermore, to verify if the Cybathlon-based training and related competition had a positive and substantial influence on the relationship between Pilot 1 and Hannes, we administered to both him and a non-runner Hannes user the same evaluation on functionality, embodiment and user experience with tests and questionnaires unrelated to Cybathlon.

## Methods

**Fig. 1:**
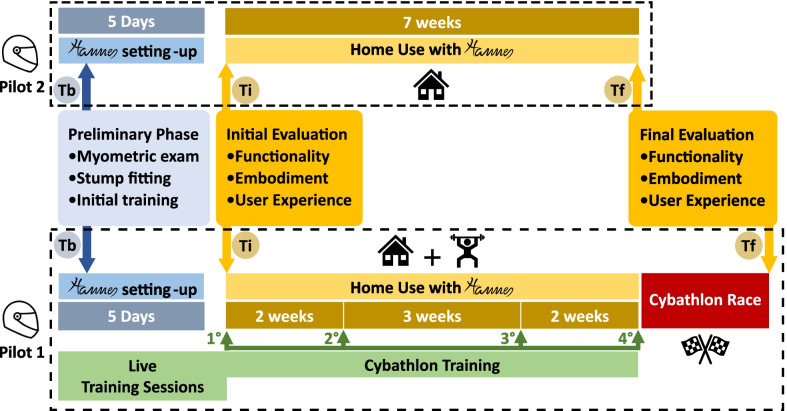
Scheme of planned activities: both pilots underwent the same Preliminary Phase (Tb) of five days for the setting up of the Hannes prosthesis and then started their seven weeks of Home Use with Hannes. At the beginning of this period, they participated to the Initial Evaluation (Ti) for the investigation of functionality, embodiment and UX with Hannes. Pilot 2 repeated the same tests and questionnaires at the end of the seven weeks in the Final Evaluation (Tf). Pilot 1 participated to the same Final Evaluation right after the Cybathlon Race, performed at the end of the same Home Use period, during which he also followed four Live Training Sessions

Two male right transradial amputees and myoelectric prosthetic users were recruited as pilots in February 2020 to form the REHAB TECH team. The first, aged 32, has a 5-years’ experience in myoelectric control with a Bebionic polyarticulated prosthesis by Ottobock, and was chosen as first pilot (Pilot 1). The second participant, age 34, is a user of a tridigital Myohand Varipuls Speed by Ottobock since 2010, and was selected as second pilot (Pilot 2). Both subjects were given the Hannes prosthetic hand and had the chance to keep it for seven weeks for Home Use. They underwent a Preliminary Phase (Tb) with a prosthesis fitting process of five days, comprising a myometric exam for the residual muscles functional state evaluation and EMG sensors positioning, stump fitting, and preliminary training, including familiarization with the Hannes prosthesis. Due to the movement restrictions caused by the COVID-19 pandemic that hit Italy and the entire world and official institutional restrictions, we were forced to train solely Pilot 1 for the Cybathlon competition. However, we decided to still let Pilot 2 use Hannes prosthesis for the predetermined Home Use period. Given his participation, for an ease of understanding, we chose to keep referring to him as Pilot 2, even if he was completely unfamiliar with Cybathlon training, competition, tasks and anything related to it. The Cybathlon Training with Pilot 1 consisted of only four Live Training Sessions of 2 days each, executed during the seven weeks of Home Use. The training started at the end of September 2020, and it was completed the day before the competition, which took place on the 12th of November. This poor preparation was due to the distance of the pilot’s residence from the training location, i.e. the Rehab Technologies Laboratory of Istituto Italiano di Tecnologia (IIT). Functionality, Embodiment and User Experience evaluation with Hannes was carried out for both pilots at the end of the Preliminary Phase and before the Home Use period in the Initial Evaluation (Ti). The same methods were repeated in the Final Evaluation (Tf) by Pilot 2 after the Home Use, and by Pilot 1 after the Cybathlon Race, occurred immediately after the same Home Use period (Fig. 1). The evaluation was carried out with tests and questionnaires unrelated to Cybathlon race, on which both pilots were not specifically trained. During the Home Use, the pilots were encouraged to use the Hannes prosthesis in all possible activities, including work, domestic and recreational contexts, as if it was their sound hand.

### The Hannes prosthetic system

The Hannes hand prosthesis is a poly-articulated myoelectric prosthetic hand. The major improvements provided by this device are the naturalness of forms, movements and orientation of the rotation axes and hand posture. Indeed, the prosthetic design was developed focusing on the anthropometry of the real human hand, both from an aesthetic and biomechanical point of view, allowing the user to perceive the device as an integral part of the body rather than a simple external tool.

**Fig. 2:**
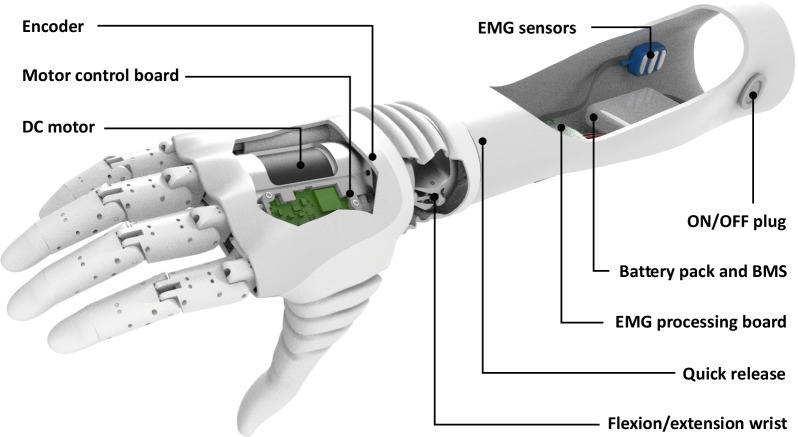
The Hannes Prosthetic System

The Hannes system contains four main components: an electric actuator (DC motor), two custom-made control boards (Motor control board and EMG processing board), two EMG sensors implementing a proportional dual-site control and a tendon-driven, underactuated transmission mechanism (Fig. 2). The embedded differential system is capable to properly offer the patient a harmonious, quick, and precise grasping behaviour. The passive Flexion/Extension flexible wrist, separately placed at the base of the prosthetic hand to guarantee system modularity, can be fixed in five different positions (two for flexion, two for extension and one for neutral position) or left free. In addition, the wrist module offers several discrete positions in Pronation/Supination with a 360° mechanical and electrical slip ring connection. Finally, a passive thumb can be locked in three positions to allow the main grasp types: lateral, power and pinch. All these features were implemented to develop Hannes as a prosthetic system uniquely similar to the real human hand, as it is detailed in reference [8].

### Cybathlon experience

#### Protocol

The Powered Arm Prosthesis Race included six consecutive tasks inspired by daily life activities, to accomplish in the minor possible time and to finish within eight minutes. Some tasks required the use of both hands and arms, simulating bimanual interactions, whereas others forced the pilot to exploit only his prosthesis. In this case, the object, or the part of it colored in blue, could exclusively be manipulated by the device. Two tasks (‘Clean Sweep’ and ‘Home Improvement’) were slightly modified, and one completely changed (‘Wire Loop’ into ‘Stacking’) after the subscription to the competition, in accordance with the new Global Edition format [12], which was designed to overcome limitations given by the pandemic crisis.

The racetrack was composed of:

1. *Breakfast task—14 points*
  Preparing a meal using kitchen tools is a fundamental activity for an autonomous living. In this task, a breakfast table must be set up by cutting bread, opening a bottle, a jam jar and a can, unwrapping a pack of sugar cube, and lighting a candle with a matchstick.
2. *Laundry task—15 points*
  This task challenged the practicality of the prosthesis during the wearing of standard clothes and its ability with fine activities such as tying shoes, closing and opening a zip, hanging a t-shirt with clothespins, and closing two blazer’s buttons.
3. *Clean Sweep task—16 points*
  This task required the ability to handle and grasp objects with different size, weight, texture and shape, which can be easily found in daily life. Eight blue objects (a pen, a plastic glass filled with balls, a USB pen, a ball, a key with a ring, a coffee mug filled with balls, a credit card, and a DVD case) must individually be moved from a table to another one, also testing the prosthetic user’s capability to maintain the grasp and the grip force during big movements. Originally, all these objects were supposed to be located in a dedicated wooden support.
4. *Home Improvement task—17 points*
  Being able to accomplish manual duties during maintenance work at home is again important for the independent living of an amputee with his prosthesis. Indeed, in this task the pilot must cut a paper with scissors, drive in a nail, and insert a light bulb in a lamp.
  In the last version, stair climbing and descending with hammer and scissors in a toolbox was removed.
5. *Haptic Box task—20 points*
  The sensory feedback implemented in a prosthetic system could improve control, acceptance, and embodiment of the prosthesis. By relying only on this feedback from the prosthesis, for example with vibrations at the socket, sounds or haptic feedback, the pilot should be able to recognize and match six different objects in shape and texture, hidden from the view and inserted in wooden boxes.
6. *Stacking task—18 points*
  To test the ability in the maintenance of a solid grasp during big postural movements of the arm and the body, in this task the pilot must flip ten blue cups and stack them into a vertical pyramid. This task substituted the old ‘Wire Loop’ one, where pilots were supposed to hold a conductive wire loop with a blue handle in a curved metal wire without touching it.

**Fig. 3:**
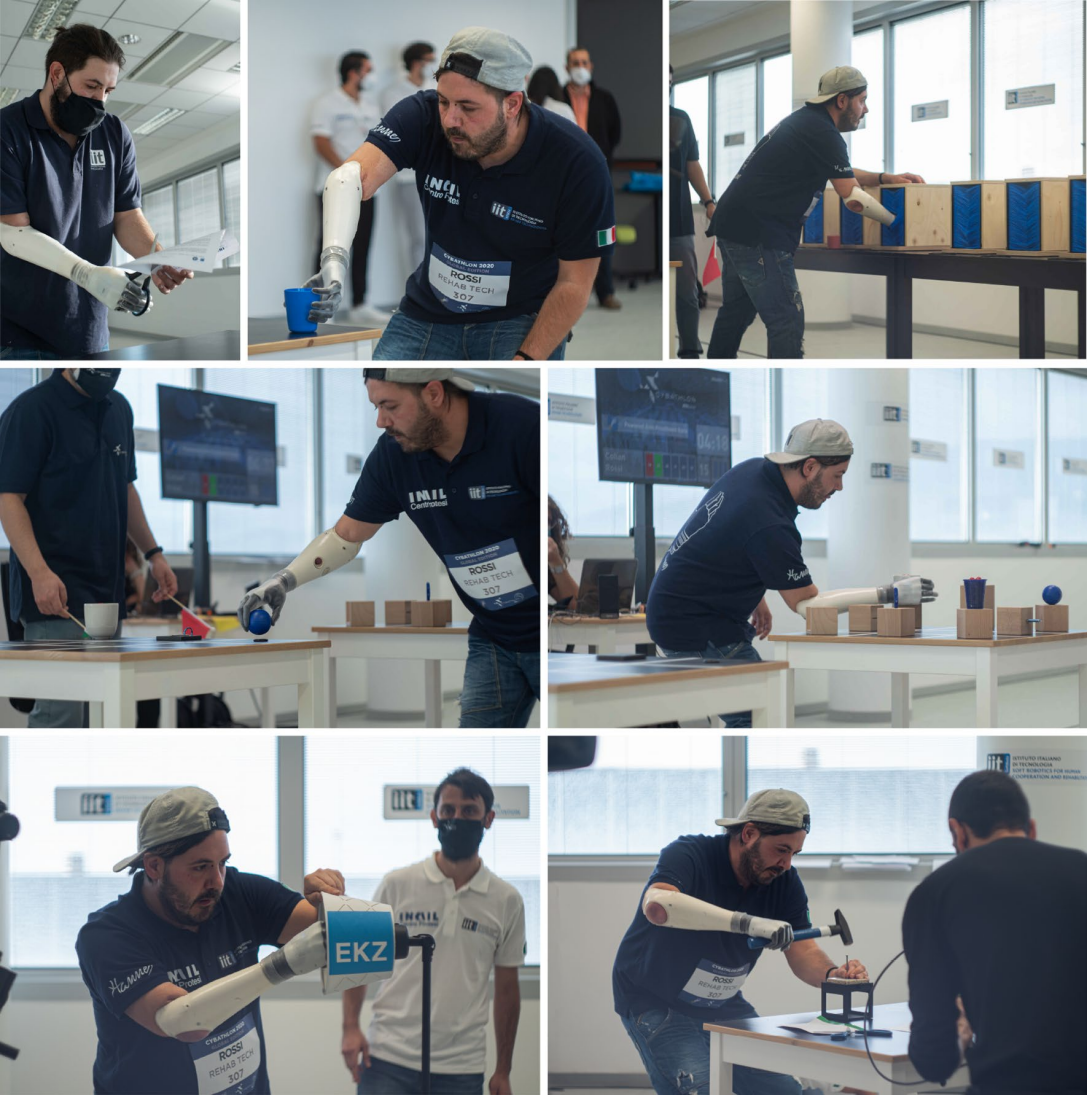
REHAB TECH Pilot 1 driving Hannes: frames of the Powered Arm Prosthesis Race of the Cybathlon 2020 Global Edition

Each team had three attempts for the race. The total score was calculated as the sum of the successful tasks, and the best attempt was considered for the final ranking. In case of equal scorings between two or more teams, the ranking was established using the total completion time, which included only successful tasks. In Fig. 3 our Pilot 1 performing some previously mentioned tasks during the official competition.

#### Training

The REHAB TECH Pilot 1’s training consisted in the repetition of each task multiple consecutive times to become familiar with the proposed activities. The most difficult tasks and specific exercises were identified, and a particular attention was given to them. During the trainings and the Home Use, Pilot 1, together with the help of the team, focused on overcoming these difficulties, searching for the right strategy to successfully complete the tasks.

**Fig. 4:**
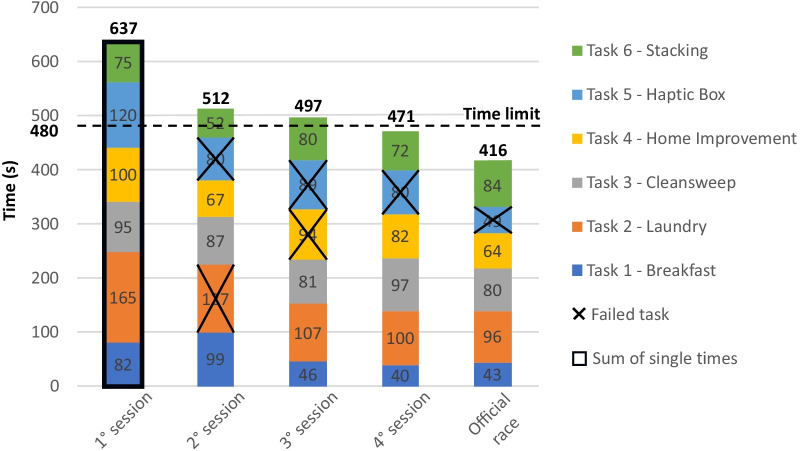
Performance evolution: the four simulated races executed during live training sessions and the performance of the official race

In the first training session each task setup was prepared one by one, and Pilot 1 repeated each task until it was accomplished. Accordingly, we collected the time of the best performance of each successful task, and during the data processing we summed these times to recreate a complete race. For this reason, this first simulated race, highlighted with a black squared box in Fig. 4, does not contain any failure. Afterwards, three other simulated races were performed at the end of each successive training session, following official rules of the competition, with the six tasks performed in a row on the racetrack. We then examined all these simulated races and the official one, reporting the time of completion of each single task, to investigate the evolution of the performances achieved during the training.

Throughout the training period we additionally collected notes and considerations about the pilot’s strategies, its performances and the type of proposed tasks and activities, which could be useful to improve the training of future Cybathlon competitions and even the practicing of an amputee with a new prosthesis.

After the competition, a further analysis was carried out to better understand Pilot 1’s results with respect to the other participants. We analysed the time of each task of the best race of each team, from which the ranking of individual task is appreciable.

### Hannes’s evaluation

The aim of the evaluation, which involved both pilots, was to assess possible progresses and improvements in the dexterity while using Hannes (from a functional point of view), its embodiment process, and user experience, focusing the attention on a possible positive impact of the Cybathlon-based training and competition in Pilot 1’s results. Both pilots executed the protocol twice (Ti and Tf) and were not trained on the proposed evaluation protocol during the Home Use.

#### Functional evaluation

The functional evaluation was executed with three standard and globally validated clinical tests, performed only with the prosthetic hand, to measure the level of dexterity:

- *Minnesota Manual Dexterity Test (MMDT)* [13], only the Placing Test
  This test evaluates the dexterity obtained with the prosthesis by measuring the time spent to reorder a set of sixty small plastic discs. These latter must be placed, starting from the corner corresponding to the amputated side (in order not to invade and obstacle the field of view), one column after another in the board below, from the top to the bottom. The score is the time required to complete the task; therefore, lower scores indicate better performances.
- *Southampton Hand Assessment Procedure (SHAP)* [14]
  This clinical test, designed to evaluate the functionality of the upper limb, measures the time spent to move forward on a board six different objects, both light and heavy, with six different grips, and the time needed to accomplish fourteen ADLs. These results are then used to calculate six indices, one for each grip, and an overall index called Index Of Functionality (IOF) (0–100), which provides an overall assessment of hand function. Scores between 95 and 100 are considered referable to the normality range. Accordingly, higher scores suggest better functionality.
- *Box and Block Test (BBT)* [15, 16]
  BBT test is composed by a wooden box divided into two compartments, separated by a wall, and by 150 wooden cubes with a lateral length of 2.5 cm. The subject must move, one by one, the maximum number of cubes from one compartment to the other one, without touching the wall, within 60 s. The final score is the total number of transferred cubes, hence higher scores manifest better dexterity.

Furthermore, two standard and globally validated questionnaires were administered with the same aim:

- *Quick Disabilities of the Arm, Shoulder, and Hand (QuickDASH)* [17]
  This questionnaire provides a general measure of the functional activities and the musculoskeletal disorders of the upper limb, validated for amputated patients. The subject must evaluate his capacity in performing eleven actions while thinking about his last week, by choosing from a 5-point scale where 1 is “No difficulty” and 5 is “I could not do it”. The final DASH score is a number between 0 and 100, and lower scores indicate better performances.
- *Orthotics and Prosthetics User Survey-Upper Extremity Functional Status module (OPUS-UEFS)* [18, 19]
  This scale is specifically designed for amputees, and it includes twenty-eight activities concerning the self-care and the usage of daily life instruments. The evaluation method consists in a 5-point scale from 4 (the subject can easily perform the task) to 0 (the subject is not able to perform the task), besides the “not applicable” choice, with the additional information about the performing of the task with or without the prosthesis. The outcomes are the percentage of usage, exploiting the number of tasks indicated to be executed with the prosthesis, and its goodness.

#### Embodiment evaluation

The embodiment is the integration of an external object into the internal body scheme: an embodied object is hence perceived as if it is part of the body itself [20]. The level of embodiment was first evaluated with postural sway, searching for potential variations, and preferably decreases, with Hannes over time. The postural balance test required the subject to be motionless standing on a force plate, with his knees straight and arms down at his sides. First, the participant had to look at an eye-level fixation point on the wall for 60 s (eyes-opened—EO—condition). Immediately afterward, he had to close his eyes and remained standing for 60 s (eyes-closed—EC—condition). The sample frequency of the data acquisition, made with the force plate (AMTI), was 1000 Hz, while the data were extracted and elaborated by a custom-made Matlab software [21] and filtered with a low-pass filter with a cut-off frequency of 20 Hz. The required parameters were calculated and generated in an Excel file as described in [21]. The programmed outputs were:

- Centre of Pressure (CoP) medio-lateral (ML) path length, calculated as the cumulative displacement in the medio-lateral direction of the CoP [22, 23];
- CoP anterior–posterior (AP) path length, calculated as the cumulative displacement in the anterior–posterior direction of the CoP [22, 23];
- CoP total path (TP) length, calculated as the cumulative displacement of the CoP [22, 23];

Furthermore, three items of an ad-hoc questionnaire (item 1, 2, 3; subsection ‘Embodiment’), inspired by the RHI questionnaires [20, 24], explored the embodiment obtained with Hannes in a subjective way and are shown in Table 1. The ad-hoc questionnaire was formulated with statements as in [25] and as the standard validated questionnaires exploited in this evaluation.

**Table 1:** Ad-hoc subjective questionnaire

Subsections	Items
Embodiment	1. When I look at the prosthesis it seems to look directly at my own hand instead of a device
	2. The prosthesis seems to be part of my body
	3. I feel the prosthesis belongs to me
System use	4. I feel I can exploit the device at its best
	5. I manage to well coordinate the two hands using them together
	6. I am immediately able to understand if an object was reachable or manipulable with the prosthesis
	7. I don’t think the device presents usage risks (that do not depend on me)
Daily life impact	8. I think I can use the device in different daily contexts
	9. The device brought positive changes in relationship with others
	10. The device usage improves my quality of life in terms of autonomy

#### User experience evaluation

The user experience is intended as the set of subjective consequences of all relationships between the user and the prosthesis [26], in term of individual perceptions, expectations, and reactions, also about aesthetics, comfort and technology acceptance [27]. User experience can be investigated through questionnaires filled out by the subjects. In our case, seven statements of the already cited ad-hoc questionnaire had the goal to evaluate these aspects and are reported in Table 1. Four of them belong to the ‘System Use’ subsection (item 4, 5, 6, 7), the remaining three (item 8, 9, 10) to the ‘Daily life impact’ one.

The ad-hoc questionnaire (Table 1) hence contained a total of 10 statements, to which assign a score within a 5-point Likert type scale, where 1 was ‘Strongly Disagree’ and 5 ‘Strongly Agree’ in order to maintain the same form of the previous validated questionnaires. In all items, an increase in the scoring means an improvement.

The user experience was also investigated with three other standard and validated questionnaires:

- *Raw NASA Task Load Index* (*Raw NASA-TLX*) [28]
  This questionnaire measures the workload perceived to assess a task or a series of performances. The perception of the workload can change, depending on the level of UX reached. It consists of 6 subjective subscales, rated within a 20-point range with 1-point steps. The raw version of the NASA-TLX, exploited in this evaluation, lacks the individual weighting of the subscales, there is evidence supporting this shortened version, which could even increase experimental validity [29]. The NASA score is calculated as a percentage, and lower scores represent better performances.
- *System Usability Scale* (*SUS)* [30]
  This scale provides a validated tool for measuring the usability of a system. It comprises 10 statements with five response options, from ‘Strongly disagree’ (1 point) to ‘Strongly agree’ (5 points). It is a very easy scale, and it can be used on small sample sizes, assuring anyway reliable results. Therefore, it can effectively differentiate usable and unusable systems. The SUS score is calculated as a number between 0 and 100, and it is considered above average when higher than 68 points, meaning that the system is usable. Hence, higher scores indicate higher usability.
- *Trinity Amputation and Prosthesis Experience Scales (TAPES),* only part 1 [31]
  This questionnaire measures the overall appreciation in using the prosthesis and its influence in the performance of ADLs. Part 1 of TAPES consists of two subscales with a 5-point rating scale regarding, respectively, the psychosocial adjustment, and the prosthetic satisfaction. The overall index is shown in the form of a percentage, and higher scores indicate greater levels of adjustment.

Results obtained in both Initial and Final Evaluation are analysed, together with the difference between the final and the initial value (Tf − Ti).

In addition, rates of improvement/deterioration of the Final Evaluation (Tf) with respect to the first one (Ti) are evaluated with the following formula:

$${T}_{f}-{T}_{i} [\mathrm{\%}]=\frac{\left({T}_{f}-{T}_{i}\right)}{{T}_{i}}\times 100$$

## Results

### Cybathlon training sessions

The results of each simulated race executed during the training period are reported in Fig. 4, showing performance progresses achieved during the training by our Pilot 1. A gradual decrease can be appreciated over time. From the first simulated race (637 s), higher than the time limit (480 s equal to 8 min) of more than two minutes and half, the total time decreased and became lower than the limit in the last training session (471 s) and in the official race (416 s). In the second simulated race Pilot 1 failed task 2 and task 5, in the third one task 4 and 5, while in the last simulated race only failed task 5, as in the official competition.

### Cybathlon official competition

The final ranking of the Cybathlon 2020 competition is shown in Table 2. Only three teams over thirteen succeeded in all tasks, obtaining the maximum score of 100 points. Our team REHAB TECH placed in the sixth place with a score of 80 points due to the error occurred in task 5 (which had the highest scoring of 20 points), and with a total time of 416 s.

**Table 2:** Final ranking of the Powered Arm Prosthesis Race of the Cybathlon 2020 Global Edition

Rank	Team	Score	Total time (s)
1	Maker Hand	100	344
2	SoftHand Pro	100	403
3	e-OPRA	100	452
4	SuperMotorica	86	390
5	BFH HuCE	86	425
6	REHAB TECH	80	367
7	x-OPRA	80	414
8	Hands On	70	319
9	Viswajyothi	51	360
10	Smart ArM	49	366
11	Touch Hand	34	252
12	CyberTum ARM	35	360
13	Imperial ARM	15	151

**Fig. 5:**
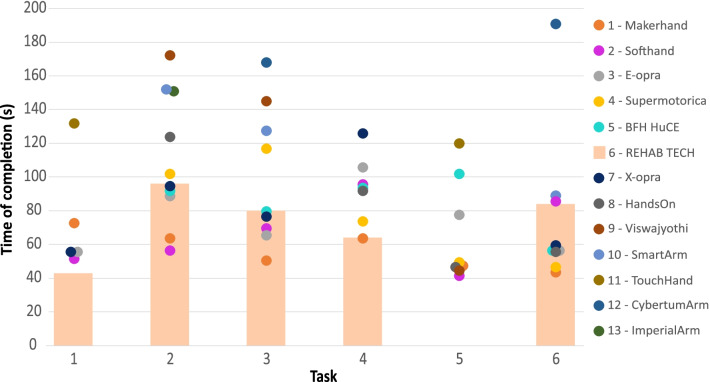
Time of completion of each team for each task: REHAB TECH’s times are enhanced and reported with light orange bars, whilst the rest of the teams’ results are shown with coloured points. Each team is correlated with a colour, as explained in the legend on the right

Figure 5 graphically shows the time of completion of each team for each task. Precisely, the main light orange bars represent our team REHAB TECH’s performance, whilst other teams’ times are reported as coloured points. No time was reported for each team if the task was failed. Lower times indicate better performance. Task 1 was completed by only six teams out of thirteen, task 2 by eleven, task 3 by 10, task 4 by 8, task 5 by 8 and finally task 6 by 10.

Our Pilot 1 had the best time performance in task 1, which was failed by many teams (seven on thirteen). Same result, but on par with the winner team Maker Hand, was obtained in task 4. The sixth and fifth places achieved respectively in task 2 and task 3 shows anyway a good mid-table result in the handling of different common objects with different grasp types. Task 5, as can be appreciated by the absence of REHAB TECH’s time of completion in Fig. 5, was failed. Only in task 6 our team REHAB TECH ranked in the second half of the ranking.

### Functionality, embodiment, and user experience evaluation

The scores of functionality, embodiment, and user experience measures of the two evaluations (Ti, Tf) for both pilots are shown in Table 3, together with the difference between the two values (Δ). In Functionality section, both pilots decreased their times in the execution of MMDT and SHAP tests (which accordingly increased the IOF), while increased the number of transferred cubes in BBT test and their questionnaires’ scores, except for the QuickDASH of Pilot 1. In the Postural balance test, which was employed to measure the embodiment, the body oscillations decreased in Pilot 1 in each investigated condition. Differently, Pilot 2’s sway increased, except in the medio-lateral direction and in the eyes-opened condition. Pilot 1’s ad-hoc questionnaire total scores increased respectively of 3 points in subsections ‘Embodiment’ and ‘System use’ and 2 points in subsection ‘Daily life impact’, whilst Pilot 2 made the same total score in the first two subsections, and slightly increased the last one (2 points more). Regarding the last validated questionnaires investigating User experience, both raw NASA-TLX scores decreased, while both SUS and TAPES ones increased.

**Table 3:** Evaluation of Hannes’s functionality, embodiment, and user experience

		Pilot 1			Pilot 2		
		Ti	Tf	Δ	Ti	Tf	Δ
Functionality							
MMDT [s]		180.0	150.0	− 30.0	196.0	179.0	− 17.0
SHAP IOF [%]		58.0	76.0	18.0	61.0	75.0	14.0
BBT [n°]		18.0	24.0	6.0	16.0	21.0	5.0
QuickDASH		11.4	11.4	0.0	13.6	11.4	− 2.2
OPUS-UEFS: usage [%]		50.0	75.0	25.0	67.9	71.4	3.5
OPUS-UEFS: goodness [%]		71.4	79.8	8.4	69,7	75.0	5.3
Embodiment: postural balance test							
TP length [mm]	EO	477.9	411.9	− 66.0	482.7	500.7	18.0
	EC	589.7	479.4	− 110.3	665.7	808.2	142.5
ML path length [mm]	EO	222.1	177.2	− 44.9	251.4	186,2	− 65.2
	EC	270.4	177.2	− 93.4	323.7	387.9	64.2
AP path length [mm]	EO	356.9	334.9	− 22.0	360.3	433.5	73.2
	EC	438.6	410.9	− 27.7	514.7	638.8	124.1
Embodiment and user experience: AD-HOC questionnaire							
Embodiment		7	10	3	8	8	0
System use		17	20	3	17	17	0
Daily life impact		13	15	2	10	12	2
User experience							
Raw NASA-TLX [%]		34.2	11.7	− 22.5	40.8	25.8	-15.0
SUS		95.0	100.0	5.0	87.5	90.0	2.5
TAPES, part 1 [%]		80.0	93.3	13.3	83.3	89.2	5.9

**Fig. 6:**
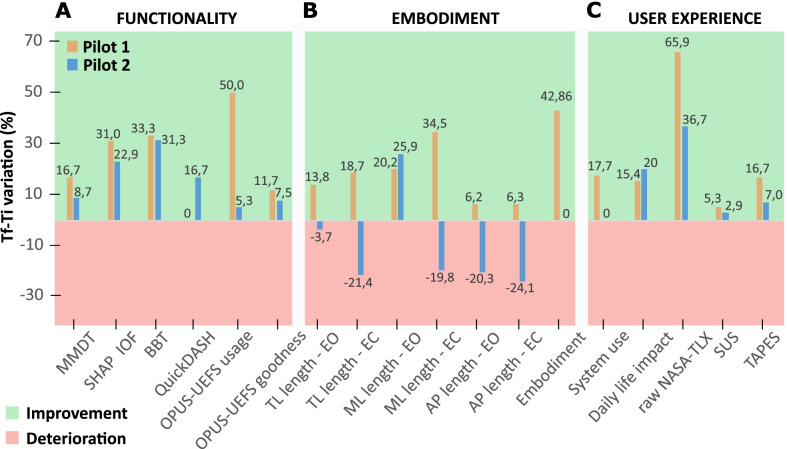
Rate of improvement/deterioration of the scores for the three investigated areas: **A** the functionality, **B** the embodiment and **C** the user experience. Positive values correspond to improvements, negative values to deteriorations

Figure 6 graphically shows the rate of improvement/deterioration of both pilots over the evaluations. Regarding the functionality, a general improvement can be observed in Fig. 6A for both pilots, however Pilot 1 exhibits overall higher percentages of improvement, especially for the OPUS-UEFS usage (50.0% against 5.3%). Only the QuickDASH score shows a small improvement for Pilot 2 and a stable result for Pilot 1.

In the postural balance test Pilot 1 improved all his parameters in each of the investigated condition, whilst Pilot 2 had an overall worsening. He only improved one variable (ML length) in one single occasion (EO), with a percentage of improvement slightly higher than Pilot 1’s, as shown in Fig. 6B. In the ad-hoc questionnaire Pilot 1 improved his total scoring in ‘Embodiment’ (Fig. 6B) and ‘System use’ (Fig. 6C) subsections, whilst Pilot 2 did not show any improvement over time. Instead, in ‘Daily life impact’ module, the percentage of improvement was higher for Pilot 2, as shown in Fig. 6C. The highest percentages of improvement in the last three validated questionnaires of the user experience belong to Pilot 1, as it can be seen in Fig. 6C.

## Discussion

### Cybathlon training and competition

As expected, a gradual improvement in the tasks’ completion time was observed with the advance of the training (Fig. 4), which allowed Pilot 1 finding the most reliable and fast strategy to accomplish each task.

The result trend followed the learning process of our Pilot 1, who learnt how to exploit Hannes and to formulate successful strategies. Some tasks and relative subtasks were performed very easily and quickly, whilst others, which initially seemed to be physically impossible, required a more intense training. No specific difficulties were found in the execution of the subtasks of task 1, since they were all bimanual exercises very similar to usual daily life activities, typically performed during the Home Use. Hence, the training was immediately focused on reducing the completion time. In contrast, the clothespins subtask of task 2 revealed to be challenging. Indeed, it was difficult to manipulate the clothespin while also having a strong-held grip in a comfortable posture. With some practice, Pilot 1 was able to identify the right strategy, which permitted a strong and safe grasp in a natural way, with him in front of the clothesline and the clothespin held between index and thumb, as performed by non-amputees. Subtasks composing task 3 were accomplished quite easily as they were performed using common grasp strategies. Task 4 was found tough in all subtasks, as only the prosthetic hand was allowed to manipulate the blue objects comprised in it: hammer, bulb and scissors. The adopted strategy exploited the edge of the table to insert thumb, index, middle and ring prosthetic fingers into the scissors’ handle to assure a firm grip and a continuous scissors’ operation for cutting the paper. The lamp subtask needed long training to find the optimal postural movements’ combination of legs, prosthetic side elbow and shoulder for the screwing of the bulb without active pronation/supination of the wrist. Instead, the hammer subtask was relatively easy, as the strong grasp and mechanical resistance provided by the prosthesis allowed nail hammering without risk of tool loosening and prosthesis rupture. As well, task 6, where only the prosthetic hand was allowed to touch blue cups, needed an intense training to overcome the absence of wrist active pronation/supination, forcing compensatory movements of the prosthetic side shoulder and the constant bending of the legs. The most difficult task was task 5, as Hannes prosthetic hand is not equipped with a haptic feedback. Hence, a safe strategy capable to assure a successful result in a very short time, and with the pressure of the competition, was not found. Pilot 1 tried to recognize the stiffness (soft or hard) of the hidden objects by slamming the prosthesis on it, listening to the sound response, and sensing the mechanical resistance of the squeezed object. The shape was instead evaluated by slipping the back of the hand on the object’s surface, looking for edges or curves.

In the official competition (Fig. 5), the peculiarity of the prosthetic hand Hannes, capable to adapt to the grasped object, and the flexible wrist module in flexion/extension helped Pilot 1 in succeeding in task 1, in which a high number of objects with different sizes and type of grasps were included. The same key role was played by Hannes in task 2 and task 3. However, both completion times were not low. This might be a consequence of Pilot 1’s approach, who gave priority to grasping precision to replicate natural hand strategies, rather than execution’s speed. For example, the tridigital grasp implemented by thumb, index and middle fingers and used for the clothespins of task 2 and the USB pen of task 3 resulted to be very natural. However, more time was required to manage this grip, as the object needed to be precisely picked with the distal phalanges. The absence of active wrist pronation/supination negatively influenced task 6, in which a flipping movement was required, leading to consistent compensatory movements and slowing down the performance. Furthermore, a small error occurred during the pyramid construction, as one cup slipped over another one and the pilot had to relocate it. The lack of active wrist pronation/supination also impacted in the bulb insertion of task 4. Nevertheless, the quick execution of the rest of the task, permitted by the strong and natural grasp of the hammer and the continuous actuation of the scissors offered by Hannes, allowed our pilot to obtain the best time, together with the Maker Hand team. The failure in task 5 was quite expected, considering Hannes is not equipped with haptic feedback system and the difficulties faced during the training to conceive an effective and reliable strategy.

Eventually, the times of completion of each task was very good or in line with the average times, meaning that Hannes performed better or similarly compared to other prosthetic devices (Fig. 5). The participation to the Cybathlon competition allowed Hannes to be compared with many other hand prostheses. Considering the position in the first half of the final ranking (6 of 13), Hannes’s performance can be considered above the average (Table 2). The absence of an active wrist module and a system capable to restore haptic feedback could be considered as limiting factors, which on our team will focus for future developments.

### Functionality, embodiment, and user experience evaluation

As expected, after almost two months of Home Use, both pilots improved their performances in all investigated areas over time, except for Pilot 2’s embodiment domain, which worsened. Moreover, it can be noted that Pilot 1 had a better learning curve and better progresses, as improvements were higher than the ones of Pilot 2.

Concerning functional clinical tests, major improvements were noticed in the most complex ones: MMDT and SHAP. In BBT test, the easiest one, both pilots improved almost in the same manner. Differently, in MMDT, which required a specific fine control for the correct inserting of the disks into the board, and in SHAP test, where several different grasps were required, higher improvements were shown by Pilot 1. This difference may be related to the additional training he faced for the Cybathlon competition, which included tasks very similar to these clinical tests. Even if the training was focused on the fast and correct execution of specific exercises and tasks, the control of Pilot 1 with Hannes seemed to improve. Differently, Pilot 2 made use of Hannes freely, without periodic training and a specific goal to achieve. This could be one of the reasons his functional improvements were lower compared to Pilot 1’s.

The difference obtained by Pilot 2 in the QuickDASH score is much smaller than the minimal detectable change (MDC) (12.85) [32], implying a not relevant clinical improvement, as the stable score of Pilot 1. These results can depend on the low specific nature of this questionnaire in evaluating amputees’ dexterity, since it provides a general measure for everyone with upper limb problems. The OPUS-UEFS scores improved for both pilots, but more for Pilot 1. He notably improved in the ‘Percentage of usage’ score, in which the difference between the final and initial value also overcomes the MDC (12.07) [19]. This parameter can be considered important because it means Pilot 1 involved his prosthesis Hannes in many more activities over time. This outcome could depend on the positive influence of the Cybathlon experience. In fact, the training and the competition seem to have improved maneuverability and functionality when compared to Pilot 2. This is confirmed by the functional tests’ outcomes reported above. Again, the training and the purpose to compete with Hannes might have motivated Pilot 1 in increasing the prosthetic usage. On the contrary, since Pilot 2 was extraneous to such experience and involvement, he may have involved Hannes in a small number of activities.

The evaluation of the embodiment, executed with an objective (postural balance test) and a subjective (items 1, 2 and 3 of the ad-hoc questionnaire) measure, seems to show an improved embodiment only for Pilot 1. Probably, the regular and increased usage of the prosthesis made by Pilot 1 due to the training had a positive impact on the embodiment domain. Overall, the results suggest that the engagement produced by the participation to the Cybathlon competition could be intertwined with the one depending on the embodiment processes. Thus, the daily usage of the system was promoted by the motivation to perform successfully during the official challenge and by the sensation that the prosthesis was part of one’s body scheme. The engagement [33] can constitute a factor promoting the embodiment of a wearable technology or can be a phase of embodiment itself thanks to the motivation to improve for the competition, in our case. However, these hypotheses will need further investigation to be evaluated. In contrast, Pilot 2 worsened the scores used to evaluate the embodiment process. Even though Pilot 1 and Pilot 2 spent almost the same total amount of time with Hannes (except for some additional days to compete the race for Pilot 1), it seems that Pilot 2 was not capable to establish a real connection with Hannes prosthesis. This could be a consequence of a basic domestic use, without the involvement driven by the motivation to prepare for a challenge. Furthermore, the difference in their results could also depend on the subject’s personality and willingness in approaching a prosthetic device as a part of the body rather than a tool: we cannot imply that Pilot 2 had a more resistive behavior, but such an interpretation suggests us to adopt personality questionnaires in future studies for checking this hypothesis.

TAPES questionnaire and subsection ‘System use’ of the ad-hoc questionnaire investigate similar situations and evaluate the perception of the subject towards the prosthesis. In our view, this represents a very important argument, and the highest improvements in relation to these two assessment tools belong to Pilot 1, as if the training and the Cybathlon competition increased such a perception. Similarly, the raw NASA-TLX scale, which measured the perceived workload while using the prosthesis, shows an improvement for Pilot 1 that is almost twice the one of Pilot 2. This may indicate that Pilot 1’s Cybathlon experience permitted to reduce the perceived workload. Overall, the higher improvements obtained by Pilot 1 suggest that the participation in the Cybathlon competition might have had a beneficial influence on his global user experience and the prosthesis impact on daily life situations. On the other hand, again, the more basic environment of Pilot 2 resulted in smaller improvements, which is in accordance with the other two evaluations.

The evaluation simultaneously conducted to the Cybathlon experience, concerning functionality, embodiment and user experience achieved with Hannes, showed overall improved scores for both pilots (except for the embodiment of Pilot 2), leading to assume that the constant and prolonged Home Use of Hannes can improve the amputee-prosthesis relationship. However, the most interesting outcome of this study is the consistent greater improvement exhibited by Pilot 1 with respect to Pilot 2. We could assume that this outcome may depend on the training, the tasks and the speed running challenge of Cybathlon 2020 competition performed by Pilot 1. Hypothetically, the engagement produced by the expectation to compete in a challenge affected the amount of daily practice of the user. Such an additional use time could have accelerated the prosthetic embodiment processes of Pilot 1, improved his speed performances, and stimulated him in finding strategies and solutions for the achievement of everyday actions, consequences not found in Pilot 2’s results, as a plausible effect of the non-attendance to the Cybathlon competition.

The embodiment phenomena are intertwined with cognitive and affective processes as in the case of motivation: motivation sustains our efforts and push us to improve our skills and performance. We hypothesize that the motivation generated by the purpose of preparing for Cybathlon competition affected the engagement of Pilot 1 in—carefully and continuously—employing the device. Such an effect helped him to establish a connection with Hannes through a steady enhancement of his skills in controlling it, directly enriching the sense of agency (a component of the embodiment).

As a limitation, only short-term benefits of the Cybathlon-based training and competition are analysed in this case study. Hence, it is not possible to state that these benefits could last in the long-term period, maintaining a consistent difference between pilots’ results in the investigated areas. Further analysis, comprising longer total time of usage and multiple follow-ups, may clarify long-term implications, evaluating if these differences persist or if both pilots reach, at some point, same level of improvement.

## Conclusion

Considering this was our first approach toward the Cybathlon experience, we can consider our sixth place a very good result. Pilot 1 learnt how to take advantage of our novel prosthetic system Hannes and was able to accomplish 5 out of 6 tasks, with execution times on average. Hannes demonstrated to be a valuable competitor, capable to perform a variety of natural grasps and to realize most of the tasks with human-like behaviors and biomimetic performances.

The results of the comparison between the user who was involved in the Cybathlon experience and the one who was not suggest that the training of a user with a prosthesis could benefit from Cybathlon’s proposed tasks and structure. The inclusion of more exercises inspired by real daily life activities, requiring their execution within a certain amount of time, could stimulate the patient’s dexterity, prosthetic embodiment and UX in a short time. It seems that timed races or trainings designed as competitions, like Cybathlon, could facilitate and even accelerate the prosthetic learning phase, decreasing the perceived workload, as possible consequences of the high developed engagement between the user and the prosthesis. This novel methodological approach should be further investigated with a precise protocol and with a consistent sample size to obtain significant results, both to improve the ADLs amputee’s performances and to better prepare our pilot for the next competition using the Hannes hand.

## Data Availability

All data generated or analysed during this study are included in this published article.

## Abbreviations

UX: User experience
ADLs: Activities of daily living
EMG: Electromyographic
R&D: Research and development
IIT: Istituto Italiano di Tecnologia
RHI: Rubber hand illusion
MMDT: Minnesota Manual Dexterity Test
SHAP: Southampton hand assessment procedure
BBT: Box and block test
QuickDASH: Quick disabilities of the arm, shoulder, and hand
OPUS-UEFS: Orthotics and Prosthetics User Survey-Upper Extremity Functional Status module
CoP: Centre of pressure
ML: Medio-lateral
AP: Anterior–posterior
TP: Total path
Raw NASA-TLX: Raw NASA Task Load Index
SUS: System Usability Scale
TAPES: Trinity Amputation and Prosthesis Experience Scales
MDC: Minimal detectable change
